# Radiology department, human factors and organizational perspectives: using action research to improve patient safety

**DOI:** 10.1186/2045-4015-2-40

**Published:** 2013-10-23

**Authors:** Osnat Tourgeman-Bashkin, David Shinar, Yoel Donchin, Ehud Zmora, Nitsa Velleman, Eugeine Libson

**Affiliations:** 1 Department of Industrial Engineering & Management, Ben Gurion University, P.O.B. 653, Beer Sheva, Israel; 2 Patient Safety Center, Hadassah Hebrew University Medical Center, Jerusalem, Israel; 3 Department of Neonatology, Soroka Medical Center, Beer-Sheva, Israel; 4 Department of Radiology, Hadassah Hebrew University Medical Center, Jerusalem, Israel

**Keywords:** Potential adverse events, Action research, Patient safety, Radiology, Organization

## Abstract

**Background:**

Action research is a participatory research method based on active cooperation between researchers and subjects. In clinical practice, action research enables active involvement of workers in developing and implementing actions promoting patient safety. This article describes a participatory action research project that was conducted in the radiology department of a tertiary care university hospital. The main objectives were: identifying potential adverse events in the department of radiology, and offering a proactive approach to improving patient safety.

**Methods:**

Phase one of the study included observing 100 patients in three units of the department and identifying potential adverse events using an observation form. According to the data obtained from the observations, multidisciplinary research teams developed and initiated, together with front-line workers, four types of interventions: ergonomic interventions in work environment design, interventions in work procedure and task design, training and guidance, and managerial interventions. Phase two included evaluation of the interventions after six months of implementation.

**Results:**

Results showed different weaknesses in each of the three radiology units tested, including incomplete medical information necessary for performing the radiological procedure, and discontinuity of care. Post-intervention observations showed a significant reduction in the prevalence of potential adverse events. At the Angiography unit, potential adverse events related to incomplete medical information dropped from 50% to 32%, and at the CT unit they dropped from 70% to 23%. At the MRI unit potential adverse events related to discontinuity of care dropped from 61% to 19%.

**Conclusions:**

The current study demonstrates the value of action research in non-hospitalizing health units and the benefits of cooperation between medical teams and human factor professionals in promoting patient safety. Methods similar to those described in the current paper are applicable to medical work teams in a broad range of practices.

## Background

Medical care professionals have recognized the importance of learning from potential adverse events as a means of minimizing medical errors and improving patient safety. “Events” in medical care may be classified into three main categories [1,2]: 1. 'Adverse events’ (AEs), namely errors with adverse outcomes; 2. 'Almost adverse events’ (AAEs, also called near misses), which are errors with no adverse outcomes or errors corrected before an AE could occur; and 3. 'Potential adverse events’ (PAEs), which are 'errors waiting to happen’, meaning failures in the system that may remain latent for a long time and cause an AE or an error with an adverse outcome (e.g. different medications stored in identical packages) [3]. PAEs can provide a useful knowledge base which enables us to study and prevent medical human errors before they cause harm.

The growing need for practical solutions to patient safety failures encouraged clinical managers in a radiology department of a tertiary care hospital in Israel to seek professional assistance from human factors professionals, in identifying ways to minimize the prevalence of failures in all aspects of work in the department. As human factors researchers, we proposed a comprehensive and proactive program for identifying failures and subsequent development of practical solutions through the use of action research methods.

Radiology, which has now evolved beyond mere X-rays, lies at the heart of medical diagnosis and treatment. Though its name has remained unchanged, many new modules which do not use X-rays are now part of this specialty, including numerous imaging procedures and machines based on different physical principles, from sound waves to magnetic fields and other computerized devices. The great progress in radiology has not been matched by the human operator’s ability to cope with the huge amount of data generated even by a single Computed Tomography (CT) or Magnetic Resonance Imaging (MRI).

Radiology is estimated to be a $100 billion per year industry in the USA alone [4] and production pressure may lead to misdiagnosis and errors [5].

The radiology field is usually one stage among many in the overall medical care process. The radiologists must obtain information from the patient’s attending physician in order to perform the correct test and reach a diagnosis. A patient arriving at the radiology department passes through a process of registration, appointment setting, examination, one or more radiology procedures (which can vary in their complexity degree, from a simple X-ray to complicated invasive procedures), and finally a diagnosis is reached. In each of these stages of the process there are many practitioners involved and each stage is a potential opportunity for human error. The cost of any error in the process can be very high, whether it is a typographical error in a patient’s name or an erroneous diagnosis.

As human factors researchers, we found that action research, with its participatory elements, is a suitable research method for addressing the problem of patient safety in health related settings. Action research is described as a research method in which the researcher works for, and with, front-line workers in their natural environment rather than undertaking external observations of front-line workers [6]. At the same time, front-line workers take an active part in the research plan and process. It is therefore important that participants understand the need to change, and agree to engage actively in all the study phases and in the change process [7]. The multidisciplinary cooperation enables comprehensive understanding of the research field, identification of real-world problems and the development of practical solutions.

As a proactive approach, action research aims to treat vulnerable points in the work environment before adverse incidents occur, and to focus on prevention of, rather than reaction to, adverse incidents. The Joint Commission has designed standards in support of patient safety and error reduction, which emphasize the advantage of proactive programs for identifying risks to patient safety and reducing medical errors [8]. The advantages of action research have made it common in health related settings. For example, researchers in a hospital in New York City conducted a 9-year worker participation project and described the way in which participatory action research was the key element of a worker-management process to improve and sustain patient safety and quality of care [9]. It resulted in the development of a wide range of intra-organizational processes, including the development of a hospital-wide labor-management committee, and departmental labor-management committees, performing multiple-union meetings and educational programs, and quarterly meetings of the council for co-leaders of the committees. These intra-organizational processes enhanced learning and the exchange of ideas, had a direct impact on strengthening and sustaining strategic alliance activities, and also helped to spread important solutions to additional departments in the hospital.

Nevertheless, action research may face several ethical issues, such as generating voluntary participation, ensuring informed consent, dealing with the implications and possible consequences of shared decision-making, anonymity and confidentiality, and resolving issues of conflicting interests of managers or existing organizational policy with the effects of action research [10-12]. Participants in action research are in fact co-researchers as well as being responsible for the changes in the researched situation. Therefore, it is necessary to involve the whole organization in the research [13].

The study objectives were:

1. To identify and examine PAEs in the radiology work environment.

2. To offer a proactive approach, which focuses on improving team work, creating a safety culture, promoting personal and managerial involvement in safety, and emphasizing error prevention rather than risk management.

3. To assess the benefits of action research as a tool for improving patient safety in non-hospitalizing health units.

## Methods

### Study procedure and tools

Being a crucial element of action research, maintaining the participatory character was the main rule of thumb in the current study. The research procedure therefore relied on the active cooperation of participants throughout all the research stages. The research procedure began with a meeting of all staff members working in the department: physicians, nurses, radiology technicians, administrative workers and unit managers. We introduced the request for professional cooperation, the study goals and its participatory element, and the necessity for active participants’ involvement in the research and in the change process.

Three radiology units were selected to participate in the study: MRI, Angiography and CT. In each unit we put together a multidisciplinary research team consisting of the unit manager, the head nurse, a technician, an administrative worker and a human factors professional. The next stage was developing, in cooperation with the teams, the most appropriate observation form to suit the needs of the units.

The team decided to examine the whole medical process - from the moment the patient enters to the unit, through his stay in the unit and until he is released from the unit. The process was divided into five main aspects:

1. Patient admission to the unit.

2. Existence of requisite medical information (mainly from the referring doctor).

3. Maintenance of homogeneous and antiseptic conditions throughout the procedure.

4. Patient release from the unit.

5. Continuity of care.

PAE was defined as any problem, noted in any of the aspects, that constituted a risk to the patient, based on the definition of PAEs mentioned earlier in the text [1-3]. The observation form contained indicators for each safety aspect. Some of the indicators were based on the experience and clinical judgment of the teams involved in each unit particular setting (indicators of aspects 1 and 4), and some of the indicators were based on previous studies (indicators of aspects 2, 3 and 5), as detailed below.

### Patient admission to the unit

The process of patient admission to radiology units usually start with patient identification. Failure to identify the patient correctly sometimes results in wrong person procedures and has been identified as a root cause of many other errors. The Joint Commission has listed improving patient identification accuracy as the first of its National Patient Safety Goals introduced in 2003, and this continues to be an accreditation requirement [14]. In a survey [15] of incidents in radiology and nuclear medicine, data on 606 incidents were reported to a central health Radiation protection services and have been analyzed and causes reviewed. The survey revealed that incorrect patient identification was the cause of 12% of the incidents in radiology and 14% of the incidents in nuclear medicine.

### Existence of requisite medical information

Medical information is a critical element in the patient care process, as incomplete medical information can lead to wrong diagnoses and incorrect treatments. Referring a patient to a medical procedure without the information required reflects inadequate pattern communication that characterizes the complex health system. Previous studies [16,17] found that poor communication is a major cause of human errors in medical systems, and 50% of human errors reported by medical staff persons were caused by failures in transferring medical information [16]. These cause delays in providing medical treatment and may be the main obstacle to human errors prevention in medical care systems. In a survey [15] of incidents in radiology and nuclear medicine, researchers found that performance of a wrong examination was the cause of 16% of the incidents in radiology, and that these were mostly due to information failures. The researchers mentioned a variety of reasons that lead to performance of a wrong examination, among them: the wrong examination may have been requested, handwriting may be misinterpreted by the radiographer, or the request may be marked for the wrong side of the body.

### The maintenance of homogeneous and antiseptic conditions throughout the procedure

Maintaining antiseptic conditions (e.g. washing hands before invasive procedures, wearing sterile clothing, sterile handling of equipment and sterile use of medical substances throughout the procedure) is important in order to prevent infections as well as other adverse events. Standardization and homogeneous work procedures are essential because they structure the medical work environment, and enable the prediction of events during medical procedure performance [18]. In a study [17] that examined the nature of human errors in intensive care units, one of the major findings was lack of standardization - tubes, fluid bags, and drugs were insufficiently marked during performing the medical procedures. The researchers found that these problems caused staff members to improvise and develop their own style of working. In addition, they created problems of identification and status assessment.

### Patient release from the unit

Releasing the patient from the radiology unit usually involves informing the patient about the procedure, and handing over instructions to the patient and to the referring department regarding continuing treatment (for example preventing infection after invasive procedure in the Angiography unit). Failures at this stage reflect problems in patient-physician communication and in the communication flow between various entities in the medical organization. In a study aimed at guiding physicians to achieve effective communication [19], researchers reviewed the benefits of effective patient-physician communication in the clinical context. They noted that during a typical patient-physician encounter, physicians make nuanced choices regarding the words, questions, silences, tones, and facial expressions they use and that these can affect the overall quality of care delivered to the patient. Effective communication in transfer of medical information from the releasing department to the hospitalizing department should also be maintained between different caregivers, as it contributes to a complete, coherent and updated knowledge base of the patient status [17].

### Continuity of care

Continuity of care enables a full and complete picture of the patient’s condition, and is necessary for coordination among different caregivers. Discontinuity in care may affect the quality of care, the patient’s cooperation with caregivers and his satisfaction with the clinical treatment. Researchers [20] in a Boston university hospital who examined the link between discontinuity of care and adverse preventable events, found an increase in the probability of adverse events in patients without continuity of care. Moreover, continuity of care is so important that they found that a tired intern with complete and detailed information regarding the patient’s condition is more likely to give the appropriate care without PAEs, than an intern who is not tired but lacks this information. Another study [21] showed that discontinuity in information transfer is linked to an increase in unnecessary laboratory tests and other examinations Table 1.

**Table 1:** Indicators of safety aspects observed at the radiology units

**Aspects of the medical procedures**	**Indicators**
**Patient admission**	■ Fully identifying the patient (name and ID)
	■ Informing the patient about the procedure
	■ Patient signing a consent form
**Medical information**	■ Information regarding medication required before procedure
	■ Information regarding any medical intolerance
	■ Information regarding important medical indicators (e.g. blood pressure)
**Homogeneous and antiseptic conditions**	■ Hand washing before procedure
	■ Wearing sterile clothing (i.e. gloves, mask)
	■ Sterile handling of equipment throughout the procedure
	■ Correct and sterile use of medical substances throughout the procedure
**Patient’s release**	■ Instructions handed to the patient
	■ Instructions to the referring department
	■ Informing the patient about the procedure
**Continuity of care**	■ Number of staff members replaced during the procedure
	■ Informing the patient when leaving the procedure
	■ Informing the next shift about important aspects of the procedure before leaving

As observers we employed three nursing students (one student in each unit) who carried out a total of 200 hours of observations on 100 patients at the three radiology units. The observers were informed about the study objectives and methods. They were trained to perform the observations and to use the observation form, in a pilot we conducted at the Angiography unit (with the research staff). The observers worked in two phases: prior to intervention and six months after the intervention was initiated. The observers received informed consent from every patient to observe the procedure, after being informed of the research objectives. All staff members also agreed to be observed. Noteworthy in particular is the fact that senior practitioners who engaged in the first phase of the observations were also part of the second observation phase. The observations did not interfere with the routine work and the observers did not interact with the patients observed nor with unit staff members while observing their work. The observers observed each patient throughout the whole medical process: starting with admission to the unit, through the medical procedure, and ending with the patient being released from the unit (on the same day).

The data collected by the observers was analyzed and presented to workers in several types of meetings: meetings with the general managers of the departments, meetings with each unit manager and meetings with each unit worker separately. In those meetings we discussed the data observed and workers were encouraged to suggest practical solutions for the problems presented. During this stage we generated several improvement activities, unique for each of the three units, according to the data obtained from the observations. The implementation of the said activities was iterative and took place over a period of six months, during which we provided workers with guidance and support. After six months of implementation we moved on to the second phase of the observations, which was performed in the same way as in Phase 1 except that we employed three different nursing students without informing them of the changes carried out after Phase 1.

As in the first observation phase, data were analyzed and presented to all workers. This final stage of the study involved drawing conclusions regarding ways of preserving the improvements and identifying possible future safety projects, of a similar nature, that could be carried out independently in the department.

### Data analyses

SPSS statistical software was employed for performing the statistical analyses and for assessing quantitative trends. Frequencies of PAEs were measured in each unit, and Chi square tests were used to test for significant differences between the three units. In order to examine the effect of the intervention on PAE rates in each safety aspect across units, we used a two-way Anova test. In the study there were two categorical explanatory variables: 1. Treatment (before/after) 2. Unit (three units), and therefore two-way ANOVA was the most appropriate analysis method for our data.

## Results

### Observations - phase 1

Out of 100 patients observed at the radiology department in phase 1 of the study, 34 were observed at the Angiography unit, 30 were observed at the CT unit, and 36 were observed at the MRI unit.

Analyses of all 100 patients observed revealed only 3 cases in which no PAEs were observed. The analyses showed that for each medical aspect observed, there was a different frequency of PAEs in each unit, and chi square tests indicated that these differences were statistically significant^a^. Table 2 presents the percentages of cases with observed PAEs in each unit separately, and the results of the Chi Square tests.

**Table 2:** Percent of patients with observed PAEs in each unit

**Aspects of the medical process**	**Angiography**	**CT**	**MRI**	**Chi square**
	**N=34**	**N=30**	**N=36**	
**Patients admission**	0%	60%	17%	33.11*
**Medical information**	50%	70%	19%	17.4*
**Homogeneous and antiseptic conditions**	47%	10%	NA	27.4*
**Patient release**	35%	0%	50%	20.1*
**Continuity of care**	0%	20%	61%	33.7*

NA-Not applicable in the unit *P<.01.

The analysis shows that “receiving medical information critical for the medical procedure” was the most problematic aspect in the Angiography and CT units. In the CT unit, unclear and/or missing information was reflected mostly in the documentation of medical sensitivities, and in missing details regarding the examination required (such as body side). In addition, the CT unit manifested quite high patient admission PAEs; this was reflected mainly in the admission of patients to the unit without their full identification by name or other personal information, which our subsequent observations suggested may be causing some patients to undergo the wrong examinations. The most common PAE in the Angiography unit was the absence of necessary information about medications and/or treatments required several hours before the procedure. The observations also showed that 23% of the patients arrived to the Angiography unit without an appropriate medical preparation necessary for the procedure (e.g., without critical information regarding sensitivities to medical substances, required blood tests and other preparations that should have been done 24 hours before the radiological procedure). Our subsequent observations suggested that some of them had to be referred back to their doctor/department. In addition, 47% of the observations at the Angiography unit recorded PAEs related to homogeneous and antiseptic conditions. This aspect is one of the most important elements in the Angiography unit, since its procedures are invasive and sterility must be maintained at all times. PAEs in this aspect were manifest mainly as inappropriate use and storage of medical equipment and drugs throughout the procedure.

At the MRI unit there were high percentages of cases with observed PAEs in various aspects related to the continuity in care. Observations showed that in over 60% of cases staff members changed during the procedure without informing each other or the patient. Patient release was also found as an aspect with a high prevalence of PAEs, mainly in releasing patients without providing them with relevant information.

Chi square tests showed that the differences in PAE incidence between units were significant. The results confirmed our understanding that each unit in the radiology department has a unique nature of the work environment which requires a specifically tailored intervention program.

### Development and implementation of the safety interventions

After analyzing the results, we set up meetings with managers, doctors, nurses and technicians, and together we developed a special safety intervention for each unit. The interventions included changes in four main aspects:

1. Ergonomic interventions in work environment design, such as changing the storage practices of medical equipment and medications and adding color coding for medical substances in use during the procedures.

2. Interventions in work procedure and tasks design, such as developing forms to ensure continuity in care, and redefining responsibilities.

3. Training and guidance: training workers in proper communication with staff members from different units and with other hospital departments.

4. Managerial interventions, such as greater involvement of managers through joint meetings with workers at all levels of the radiology department, in order to review PAEs and to discuss patient safety issues.

The intervention was implemented over a six months period in all three units of the radiology department.

Table 3 presents examples of PAEs and specific interventions undertake to reduce their incidence.

**Table 3:** PAEs and interventions applied to reduce their incidence

**PAE**	**Intervention**
Missing critical medical information required before procedure at the angiography unit.	Before the morning shift, nurses examined all cases of the day and called the relevant referring doctor to complete any missing information.
	Medical staff from the angiography unit had meetings with the medical staff of the major referring departments in order to demonstrate the unit work and the importance of clear and complete medical information.
At the end of the procedure, not all patients received clinical instructions.	A special form was designed, in several prevalent, languages, which contained instructions for patients.
Instructions were given orally.	
Typists of medical records made typing errors in diagnoses because doctors’ handwriting was not always clear.	Typists received lecture and guidance, and were instructed to call doctors in case of unclear handwriting.
Discontinuity in care at the MRI unit.	The MRI unit medical staff was lectured and given guidance regarding the importance of continuity in care and ways to maintain it.
	A special form was designed, requiring written relevant medical information and signature on the document before leaving the shift.
Inappropriate use and storage of medical equipment and drugs.	A senior pharmacist examined all three units and formulated recommendations for appropriate storage of drugs and equipment.
	Special color-coded stickers were designed in order to distinguish different drugs while performing the procedure.

### Observations - phase 2

Phase 2 was conducted in order to evaluate the effectiveness of the interventions in reducing PAE rates at the radiology units. Three nursing students (different from those employed in Phase 1) observed 100 patients for a total of 200 hours.

Similar to the findings for Phase 1, Phase 2 analyses of all 100 patients observed revealed only 3 cases in which no PAEs were observed. The analyses also revealed reductions in the frequencies of observed PAEs in each unit. Table 4 presents the frequencies of cases with observed PAEs in each unit separately and the results of five 2-way Analyses of Variance of Treatment (2- Before versus after) x Unit (3 - MRI, Angiography and CT) that was conducted in order to examine the significance of the effect of the intervention on PAEs rates (Degrees of freedom -2).

**Table 4:** Percent of patients with observed PAEs in each unit before vs. after intervention

**Aspects of the medical process**	**Angiography**		**CT**		**MRI**		**2 way**
	**N=34**		**N=30**		**N=36**		**ANOVA**
	**Before**	**After**	**Before**	**After**	**Before**	**After**	
**Patients admission**	0%	0%	60%	40%	17%	19%	1.2
**Medical information**	50%	32%	70%	23%	19%	8%	16.9*
**Homogeneous and antiseptic conditions**	47%	44%	10%	3%	NA	NA	0.48
**Patients release**	35%	24%	0%	0%	50%	11%	11.9*
**Continuity of in care**	0%	0%	20%	10%	61%	19%	13.1*

NA-Not applicable in the unit *P<.01.

The analyses showed that the most problematic aspects in each of the three units were improved after six months of intervention. At the Angiography unit frequencies of observed PAEs in medical information were reduced dramatically from 50% to 32%, and at the CT unit they were reduced from 70% to 23%. A dramatic reduction in observed PAEs was found at the CT unit as well, where PAEs related to the process of patient admission declined from 60% to 40%.

At the MRI unit there was high percentage of cases with observed PAEs related to continuity of care, wherein 61% of the cases staff members changed during procedure without informing each other or the patient. Following the intervention, the frequency of these PAEs dropped from 61% to 19%.

## Discussion and conclusions

The purpose of this study was to identify and reduce PAEs in radiology units, using proactive action research. The study found different pre-intervention weaknesses in each of the three radiology units studied, reflecting the fact that different environments, tasks, and work habits are involved in each unit, even though they are all grouped under the department of radiology. As a result, different safety interventions were required to suit the unique needs of each of the units.

One of the most common PAEs was related to incomplete medical information necessary for performing the radiological procedure. 45% of the patients observed in the units arrived there with incomplete medical information which was required for performing the procedure. This finding illustrates the inadequate communication between different units in the complex medical system, which may evolve from the lack of agreed upon and standard instructions regarding the transfer of medical information among various elements in the organization. The aspect of medical information failures as a cause of adverse events is evidence-based and constitutes high risk to the patient. In the current study, PAEs caused by information failures were reduced significantly following implementation of the safety intervention (at the Angiography unit from 50% to 32%, and at the CT unit from 70% to 23%).

Another major PAE found in the study was related to discontinuity of care. More than half of the medical staff members at the MRI unit changed during the procedure without transferring full and complete relevant medical information to their incoming colleagues. The aspect of discontinuity of care as a cause of adverse events is also evidence-based and constitutes a high risk to the patient. Discontinuity of care can lead to failures in the information transfer process which is an essential stage especially in medical processes involving multiple caregivers among whom communication is vital (e.g. referring doctors, referring department, radiographer). In the current study, PAEs caused by discontinuity of care were reduced significantly following implementation of the safety intervention (at the MRI unit from 61% to 19%).

The results showed a significant effect of the safety intervention on the aspect of patients’ release from the unit. At the angiography unit, PAEs caused by failures in patients’ release from the unit were reduced from 35% to 24%, and at the MRI unit from 50% to 11%. In the current study, failures in the stage of releasing the patient from the unit included mainly problems in transferring information to the patient and/or to the referring department. Patient-physician communication failures as well as communication failures among caregivers, can lead to medical adverse events with high risk to the patient. In the current study, as appear from the observations these failures apparently caused patients suffered from infections after invasive procedures at the Angiography unit due to lack of information and instructions regarding continuing treatment. In addition, these failures apparently caused repeated unnecessary radiological procedures.

In two of the aspects examined in the current study there was no significant effect of the safety intervention implemented - patient admission to the unit, and maintaining homogeneous and antiseptic conditions throughout the procedure (even though most of the PAEs related to these aspects were reduced following intervention). One of the reasons some of the aspects changed more than others, may be inherent in the fact that our safety intervention was limited in its implementation period and in its ability to create a deep change in the culture of safety. Those aspects which changed less than the others (aspect 1 and aspect 3) may be aspects for which change requires a more sustained intervention. Efforts to improve patient safety should also involve strategies to improve safety culture by changing workers’ attitudes and perceptions towards the accountability for patient safety issues.

Above all, the current study demonstrates the value of action research in non-hospitalizing health units as the radiology units. Action research has become a popular style of research in medical practice, as it enables the examination of medical issues with quantitative as well as qualitative tools, while enabling interactive studies which focus on action itself rather than just observing an action [22]. In the current study, action research led to a high degree of participants’ involvement and cooperation. Staff members from all levels of the department were actively involved in developing the safety interventions and in their implementation. Once implementation began, the staff also received continuous feedback about intermediate results. Working with staff members through all stages of the study enabled practical evaluation in the direct context of the radiology work environment and has probably increased the likelihood that the improvement will continue.

The study was limited in several ways. First, the data collection tool we used was field observations, a tool subject to reliability and validity problems and also to a Hawthorne effect – subjects may improve an aspect of their behavior as a response to the fact that they know they are being studied [23]. Moreover, in the two observation phases there were different observers and different patients and procedures. There was also no control group for comparison.

The difficulty in isolating variables in multi-factorial system changes is a challenge to health organizational research. Some of these problems can be minimized in future studies of this type by employing several observers in each unit, collecting a greater number of observations and testing for interobserver reliability.

In addition, we did not attribute weights to the different PAEs according to their severity and potential impact on patient safety. Classifying PAEs according to their potential severity could have broadened the findings, but testing and using a method for severity classification was beyond the scope of our study.

We also did not relate the reduction in PAEs to a reduction in actual adverse events. The availability of information on adverse events depends on the existence of a good and transparent reporting system, which unfortunately did not exist in the units examined in our study. Nevertheless, we believe that a decrease in PAEs prevalence will inevitably lead to a decrease in the occurrence of adverse events.

The study’s implications at the departmental level are continuing until today. The department won a safety award a year after completing the study and it constitutes a model for the role of participatory action research as a tool for improving safety culture and, as a result, improving patient safety. The radiology units’ medical staff learned to create more positive communications, managers learned to communicate better with workers and to actively involve them in safety programs.

The positive outcomes of the study influenced all units in the hospital due to the fact that part of the intervention program included meetings with medical staff of major referring departments, in order to demonstrate the radiology units’ work and to emphasize the importance of clear and complete medical information (see also Table 3). The study’s outcomes from the first phase were presented to the hospital managers in order to generate involvement and joint work with management leaders. This step resulted in a greater awareness of the contribution of managers to safety culture, and created a positive effect that was spread to other departments in the hospital.

Furthermore, at the generic level, the study’s conclusions are valid all over the scope of medicine, as it is a proactive participatory process. Directors of other medical settings can use similar proactive processes in order to develop suitable interventions promoting patient safety.

At the national level, the study’s results suggests that accountability for quality and safety of medical processes should concern staff members at all levels of the medical organization, including managers at the national levels, and should be treated as an optimization problem of the whole healthcare system. Patient safety can be promoted by the use of proactive approach such as action research, which contributes to workers empowerment, to safety culture and to open communication and in turn, influence patient safety. Many proactive approaches do not necessarily depend on allocating financial budgets, but mainly on understanding the importance of transparency, multidisciplinary cooperation and open communication in healthcare systems.

The study demonstrates the benefits of the multidisciplinary cooperation between human factors professionals and medical practices. As reflected in the study results, human factors principles can be useful in creating a comprehensive safety management intervention that will promote patient safety through adapting systems and work environment to individual capabilities.

### Endnote

^a^It is important to note that we assigned equal weight to each aspect of the medical process observed, although the PAEs may differ in importance. However, the ethical debate regarding the clinical importance of each PAE was beyond the scope of the study.

## Competing interest

The authors declare that they have no competing interest.

## Authors’ contributions

T-BO carried out the study as part of her PhD thesis. She was responsible for developing the conception and design of the study, acquisition of the data, statistical analysis and interpretation of the data and wrote the manuscript. SD has made substantial intellectual contributions to conception and design of the study, generally supervised the study and has been involved in revising the manuscript. DY has made substantial intellectual contributions to conception and design of the study and to interpretation of the data, supervised the study and has been involved in drafting the manuscript. ZE participated in the conception and design of the study. VN participated in the design and coordination of the study and acquisition of the data. LE participated in the design and coordination of the study and generally supervised the study. All authors read and approved the final manuscript.

## Authors’ information

O. Tourgeman-Bashkin is a lecturer at the Azrieli College of Engineering in Jerusalem.

D. Shinar is the George Shrut Professor of Human Performance Management in the Department of Industrial Engineering and Management at the Ben-Gurion University of the Negev.

Y. Donchin is the Head of the Patient Safety Unit, Hadassah Hebrew University Medical School, Jerusalem.

E. Zmora is the Head of the Department of Neonatology, Soroka Medical Center, Faculty of Health Sciences, Beer-Sheva.

E. Libson is the Head of the Department of Radiology and Medical Imaging, Hadassah Hebrew University Hospital, Ein-Karem, Jerusalem.

N. Velleman is the Administrator, Hadassah Hebrew University Hospital, Mount Scopus, Jerusalem.
